# P-745. Prevalence and Characteristics of Rubella Virus-Associated Granulomas: Results from a National Multicenter Study

**DOI:** 10.1093/ofid/ofaf695.956

**Published:** 2026-01-11

**Authors:** Nicole Boswell, Alexa G Ries, Mariah Estill, Misha Rosenbach, Bridget Shields, Beth Drolet, Megan Noe, Karolyn Wanat

**Affiliations:** Medical College of Wisconsin, Milwaukee, Wisconsin; Medical College of Wisconsin, Milwaukee, Wisconsin; Medical College of Wisconsin, Milwaukee, Wisconsin; Hospital of the University of Pennsylvania, Philadelphia, Pennsylvania; University of Wisconsin School of Medicine and Public Health, Madison, Wisconsin; University Wisconsin, Madison, Wisconsin; Brigham & Women's Hospital, Boston, Massachusetts; Medical College of Wisconsin, Milwaukee, Wisconsin

## Abstract

**Background:**

Rubella virus (RuV), a single stranded RNA virus, has detrimental effects in pregnancy due to miscarriage and congenital rubella syndrome. RuV was declared eliminated from the United States in 2004 due to successful implementation of the combined measles, mumps, and rubella (MMR) vaccine.^1^ Despite this eradication, rubella virus (RuV)-associated granulomas have been reported in the literature but are limited to case reports and series, with no reported prevalence rate.^2^ This study aims to assess the prevalence of cutaneous RuV granulomas and to further characterize the disease.

**Table 1:**
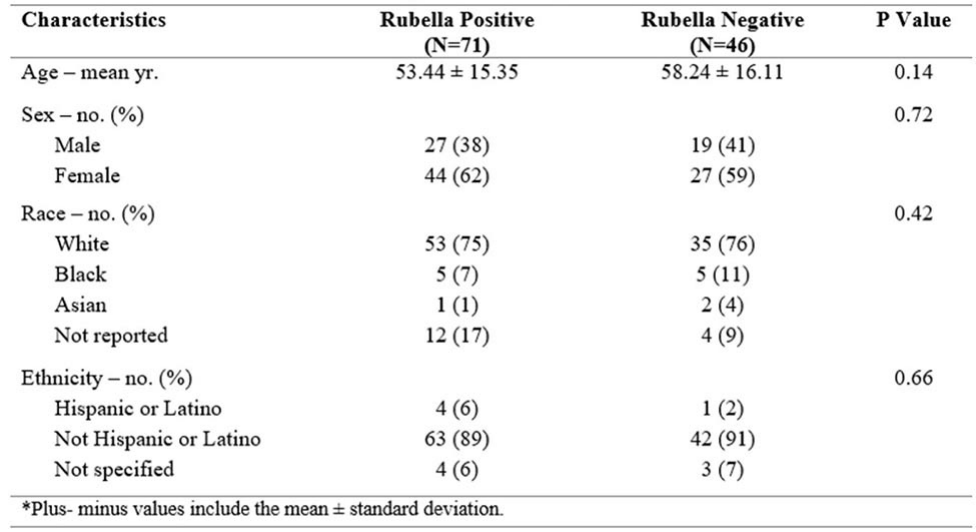
Characteristics of patients by rubella status

**Table 2:**
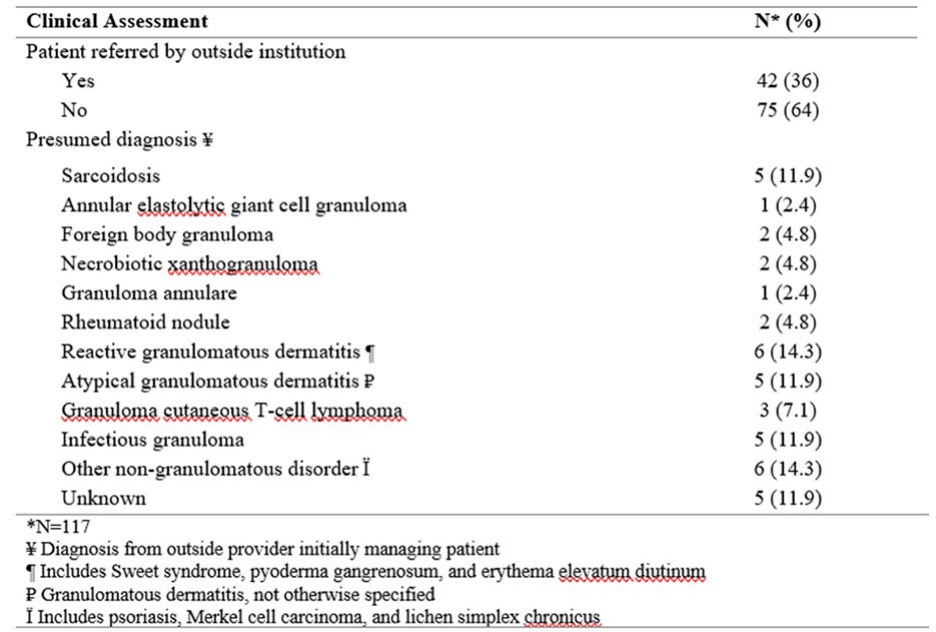
Granulomatous disease presentation and referral diagnosis

**Methods:**

A multicenter retrospective cohort study of adult patients with atypical granulomas on histopathology, defined as suppurative granulomatous inflammation without an infectious cause or a granulomatous pattern without a specified diagnosis, were included. RuV immunostaining was performed on paraffin-embedded cutaneous tissue by the Centers for Disease Control and Prevention (CDC). Demographics and record review were collected.

**Results:**

Of 117 patients, 71 were RuV-positive (61%) with a mean age of 53.44 ± 15.35; 61% were female (Table 1). RuV-positive lesions predominantly occurred on the extremities (80%, p=0.04) followed by head/neck (7%) and trunk (7%). Known immunocompromised status was not more prevalent in RuV-positive cases compared to RuV-negative (p=0.31). A third of cases came from outside referrals with a presumed diagnosis of both infectious and noninfectious etiologies (Table 2). Of the RuV-positive cases, 77% received one or more therapies. Biologics were used for 27% of patients, with TNF inhibitors most commonly used (72%).

**Conclusion:**

In this study, a high number of atypical cutaneous granulomas are positive for RuV (61%) with a predilection for the extremities. Atypical granulomas with extensive and unrevealing results should prompt testing for RuV. Future directions include thorough evaluation of patients, potential shedding into the environment and identification of best therapeutic interventions, including effective anti-virals against RuV. There is a need for prospective analysis to determine if RuV is vaccine-derived or wild type to further support public health interventions.

**Disclosures:**

Misha Rosenbach, MD, Johnson & Johnson: Advisor/Consultant|Merck: Advisor/Consultant|Novartis: Advisor/Consultant|Priovant: Advisor/Consultant|Priovant: Grant/Research Support Bridget Shields, MD, Priovant Therapeutics: Advisor/Consultant|UpToDate: Chapter Author Beth Drolet, Md, Arkayli: Board Member|Arkayli: Owner|Arkayli: Ownership Interest Megan Noe, MD, MPH, MSCE, Boehringer Ingelheim: Advisor/Consultant|Boehringer Ingelheim: Grant/Research Support|Bristol Meyer Squibb: Grant/Research Support|Sanofi: Employee|Takeda: Advisor/Consultant

